# Predictive Quantitative Structure–Activity Relationship Modeling of the Antifungal and Antibiotic Properties of Triazolothiadiazine Compounds

**DOI:** 10.3390/mps4010002

**Published:** 2020-12-27

**Authors:** Michael Appell, David L. Compton, Kervin O. Evans

**Affiliations:** 1 USDA, Agricultural Research Service, National Center for Agricultural Utilization Research, Mycotoxin Prevention and Applied Microbiology Research Unit, 1815 N. University St., Peoria, IL 61604, USA; 2 USDA, Agricultural Research Service, National Center for Agricultural Utilization Research, Renewable Product Technology Research Unit, 1815 N. University St., Peoria, IL 61604, USA; david.compton@usda.gov (D.L.C.); kervin.evans@usda.gov (K.O.E.)

**Keywords:** antifungal, antimicrobial, food safety, machine learning, mycotoxin

## Abstract

Predictive models were developed using two-dimensional quantitative structure activity relationship (QSAR) methods coupled with B3LYP/6-311+G** density functional theory modeling that describe the antimicrobial properties of twenty-four triazolothiadiazine compounds against *Aspergillus niger*, *Aspergillus flavus* and *Penicillium* sp., as well as the bacteria *Staphylococcus aureus*, *Bacillus subtilis*, *Escherichia coli*, and *Pseudomonas aeruginosa*. B3LYP/6-311+G** density functional theory calculations indicated the triazolothiadiazine derivatives possess only modest variation between the frontier orbital properties. Genetic function approximation (GFA) analysis identified the topological and density functional theory derived descriptors for antimicrobial models using a population of 200 models with one to three descriptors that were crossed for 10,000 generations. Two or three descriptor models provided validated predictive models for antifungal and antibiotic properties with *R*^2^ values between 0.725 and 0.768 and no outliers. The best models to describe antimicrobial activities include descriptors related to connectivity, electronegativity, polarizability, and van der Waals properties. The reported method provided robust two-dimensional QSAR models with topological and density functional theory descriptors that explain a variety of antifungal and antibiotic activities for structurally related heterocyclic compounds.

## 1. Introduction

Quantitative structure–activity relationship (QSAR) studies identify key descriptors and properties of structurally related molecules that are correlated to biological activities based on the concept that similar compounds have similar activities [1,2]. This information can be used to develop predictive models for the design and evaluation of new antimicrobials or to economically screen libraries of compounds for antimicrobial properties in silico [3,4]. The resistance of pathogenic fungi and bacteria to popular antifungals and other antimicrobials negatively impacts public health throughout the world and spurred recent calls for the development of new antimicrobials [5,6]. Historically, heterocyclic compounds have been widely investigated for favorable biological activities resulting in extensive structure–activity relationship studies, and several classes of heterocyclic compounds have been popular leads for antimicrobial compounds [7,8,9,10]. In addition, phenolic compounds, including components of essential oils, exhibit a variety of antimicrobial properties [11,12]. Furthermore, it has been demonstrated that the efficacy of antimicrobial compounds could be enhanced through synthetic modification [13]. To this end, we report a convenient method to develop QSAR models based on topological descriptors and density functional theory derived molecular orbital properties that is capable of predicting antifungal and antibiotic activity against *Aspergillus* species, *Penicillium* species, Gram-positive *Staphylococcus aureus* and *Bacillus subtilis*, and Gram-negative *Escherichia coli* and *Pseudomonas aeruginosa* [10].

This method has previously been applied to several classes of molecules to elucidate the contributions of chemical structure to diverse activities. The method could evaluate the cytotoxicity, phytotoxicity, and detection reliability of structurally related trichothecene mycotoxins [14]. The method was recently applied to evaluate the antifungal properties of phenolic compounds against six types of mycotoxin-producing fungi [12].

The method follows a general QSAR procedure.

- Build structures;
- Calculate density functional theory properties and descriptors;
- Calculate 1D and 2D descriptors;
- Create a training set and test set;
- Apply the genetic function approximation method for descriptor identification;
- Develop mathematical models using suitable descriptors;
- Evaluate models using internal validation and external validation.

Quantum chemical descriptors were calculated to gain insight into the quantum chemical properties of the compounds used in this study. In addition, these quantum chemical descriptors provide information on the range of electronic properties of the compounds used in these models. This method is evaluated for its ability to predict inhibition activities of a class of structurally related heterocyclic compounds against a variety of important fungi, Gram-positive bacteria and Gram-negative bacteria.

*Aspergillus* species include *Aspergillus niger*, a common mold associated with commodity spoilage, high volume citric acid production, and cosmeceutical preservation [15,16]. Certain strains of *Aspergillus niger* produce the carcinogenic mycotoxin ochratoxin A [16]. *Aspergillus niger* has been considered a contributor to aspergillosis lung disease; however, aspergillosis is most frequently caused by the co-occurrence of more toxic species of *Aspergillus* genera, with *Aspergillus fumigatus* being the primary cause [17]. The second most common cause of aspergillosis lung disease is *Aspergillus flavus*, a primary producer of a wide variety of mycotoxins, including the highly carcinogenic aflatoxins [18]. The most important mycotoxins produced by *Penicillium* species include ochratoxins and patulin [19]. Patulin exposure to children is a concern due to its occurrence in apple-based products that are marketed toward children. *Staphylococcus aureus* is a Gram-positive bacterium associated with food poisoning and infectious diseases of the respiratory system and skin [20]. The resistance of certain strains of *Staphylococcus aureus* for common antibiotics is an emerging problem, including MSRA (methicillin-resistant *Staphylococcus aureus*) [21]. Certain strains of *Escherichia coli* are associated with food poisoning, and *Pseudomonas aeruginosa* is a plant and animal pathogen [10].

In this paper, we demonstrate the application of QSAR methodology to a series of triazolothiadiazine derivatives to gain insight into the contributions of electronic and chemical properties to seven different antifungal and antibiotic activities. The predictive models developed demonstrate the method and serve as tools to develop new antimicrobial compounds based on the triazolothiadiazine structure. This approach has proven successful for chalcones, phenolic compounds, coumarin derivatives, and other heterocyclic compounds [12,14,22].

## 2. Materials and Methods

### 2.1. Date Set

The dataset consisted of 24 triazolothiadiazines with biological activities reported in the scientific literature against *Aspergillus niger, Aspergillus flavus*, *Penicillium species*, Gram-positive *Staphylococcus aureus* and *Bacillus subtilis*, and Gram-negative *Escherichia coli* and *Pseudomonas aeruginosa* [9,10]. The antifungal activities were characterized by as % mycelial growth inhibition (%MGI). The antibiotic activities are reported as minimum inhibitory concentration (pIC_50_) and diameter of growth inhibition zone (DGI) [10].

### 2.2. Quantum Chemistry

Quantum chemical studies on the triazolothiadiazines were carried out using ChemAxon Marvin Suite, HyperChem v.8.0.10, OpenBabel, Mold2, Spartan ’18, and BuildQSAR software [23,24,25,26,27]. Structures were built using the ChemAxon MarvinSuite for 2-dimensional structures and HyperChem software for 3-dimensional structures. File types were converted using OpenBabel software. Quantum chemical calculations were conducted using Spartan ’18 software with the PM6 semi-empirical method and B3LYP/6-311+G** density functional theory method. Initial geometry optimization calculations were conducted using the PM6 semi-empirical method to efficiently obtain better starting geometries for the more costly geometry optimization calculations using density functional theory. All results reported in this manuscript are on fully optimized geometry structures using the B3LYP/6-311+G** density functional theory method with default optimization parameters in Spartan ’18. The properties of the highest occupied molecular orbitals (HOMO) and lowest unoccupied molecular orbitals (LUMO) were used to calculate quantum-based descriptors following previously published procedures [12,14,28,29,30].

### 2.3. Training and Test Sets

Compounds were separated into a training set and a test set based on established procedures to test the models over a range of activities. It has been shown that the rational design of the test set and training set supports better model development [31]. Compounds with maximum or minimum antimicrobial activities for any of the fungi or bacteria were included in the training set. The test set was derived from the remaining compounds to represent a spread of the activities. The training set for *Aspergillus niger, Aspergillus flavus*, *Penicillium species*, *Staphylococcus aureus,* and *Bacillus subtilis* contained 19 compounds. The training set for *Escherichia coli* and *Pseudomonas aeruginosa* had 12 compounds. The test set included 5 compounds.

### 2.4. QSAR Study

Molecular topological descriptors (777) were calculated using Mold2 bioinformatics software provided by the FDA [26]. In addition, 8 quantum chemical descriptors were added based on the B3LYP/6-311+G** density functional theory results. These density functional theory descriptors were calculated following previously reported procedures [12,14,28,29,30]. QSAR models were built and analyzed using BuildQSAR software. Descriptor selection was based on genetic function approximation (GFA) analysis on values that were centered on the mean and scaled [32]. One, two, and three descriptor models were developed using GFA analysis on populations of 200 models over 10,000 generations. Models with cross-correlations values exceeding 0.6 were not considered. The best models were identified based on the highest leave, one out cross-validation score (*Q*^2^*_LOO_*). Only models without outliers are reported. Outliers are defined as compounds that have a difference between the observed activities and calculated activities that exceeds two standard deviations. Models were validated internally by the leave one out cross-validation score (*Q*^2^*_LOO_*) and externally by the correlation coefficient squared of the test set (*R*^2^*_ext_*).

## 3. Results and Discussion

### 3.1. Chemical Structure

The chemical structures and biological activities of the triazolothiadiazines investigated in this study are shown in Table 1 [10]. These structures differ in the substituents at R1 and R2 sites and the degree of saturation of the cyclopentane ring. The substituents at the R1 site are either a hydrogen or methyl group. The substituents at the R2 site are hydrogen, methyl, ethyl, propyl, isopropyl, and phenyl groups.

Compounds **1a**–**1l** have a saturated cyclopentane ring. Compounds **2a**–**2l** possess an unsaturated cyclopentane (cyclopent-[1,2]-ene) ring, which expands the conjugated double bond system across the cyclopentane ring. Compounds **1a**–**1f** and **2a**–**2f** possess a hydrogen at the R1 position. Compounds **1g**–**1l** and **2g**–**2l** possess a methyl group at the R1 position. Overall, these compounds are highly structurally related to varying substituents and ideal for QSAR analysis.

**Figure 1:**
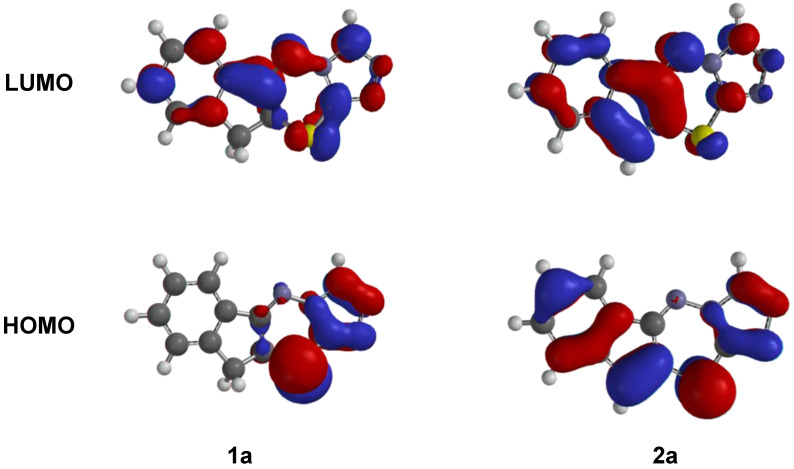
Molecular orbitals of triazolothiadiazine compounds **1a** and **2a** calculated on structures geometry optimized using B3LYP/6-311+G** density functional theory.

The B3LYP geometries of compounds **1a**–**1l** and **2a**–**2l** are shown in Figure S1 (Supplementary Material). The chemicals share remarkably similar three-dimension structures. The frontier orbital properties of the triazolothiadiazine derivatives are shown in Figure 1 for compounds **1a** and **2a**. Frontier orbitals for **1a**–**1l** and **2**a–**2l** are shown in Figure S2 (Supplementary Material). It is interesting that the degree of saturation on the cyclopentane ring has a significant influence over the position of the highest occupied molecular orbital (HOMO) and the lowest unoccupied molecular orbitals (LUMO). Compounds with saturated cyclopentane ring, **1a**–**1l**, have their HOMO orbitals covering only part of the triazolothiadiazine, as represented in **1a** in Figure 1. In contrast, compounds with an unsaturated cyclopentane ring, **2a**–**2l**, have their frontier orbitals (HOMO and LUMO) spanning the length of the triazolothiadiazine structure, as represented in **2a** of Figure 1. Frontier orbital values can be used to calculate the properties of the compounds (see Table 2).

The properties and energy levels of the HOMOs and LUMOs have valuable information on the electronic structure of compounds. Specifically, the HOMO and LUMO energy values approximate the ionization potential and electron affinity, respectively [30]. Based on this association, several descriptor properties can be calculated to shed light on the electronic properties of the triazolothiadiazine compounds [28,29,30]. The values ε_HOMO_ and ε_LUMO_ can provide bandgap energy, Δε. The chemical potential, μ, varies between -4.28 to −4.75. The electronegativity, *χ*, varies between 4.28 to 4.75. The hardness, *η*, falls between 1.45 and 2.13 eV. Softness, *σ*, provides information on the electron-accepting properties and is the inverse of hardness (values between 0.476 to 0.683 eV^−1^). A valuable descriptor calculated from the frontier orbitals is the electrophilicity index, *ω* (4.57 to 7.31). Compounds **1a**–**1l** possess larger band gaps and hardness values and lower electrophilicity index compared to compounds **2a**–**2l**. This suggests that the added double bond in compounds **2a**–**2l** increases the electrophilicity.

### 3.2. QSAR

**Figure 2:**
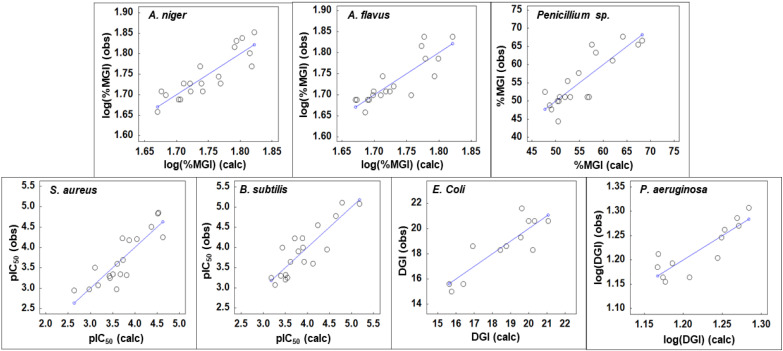
Observed vs. predicted plots of antifungal and antibiotic activities for triazolothiadiazines against *Aspergillus* niger, *Aspergillus* flavus, Penicillium sp., and *Staphylococcus aureus*, *Bacillus subtilis*, *Escherichia coli* and *Pseudomonas aeruginosa*. Several validated quantitative structure activity relationship (QSAR) equations were developed to describe the antifungal and antibiotic activities of triazolothiadiazines, and the best equations and their topological descriptors are given in Table 3. QSAR models **X1**, **X2**, **X3**, **X4**, and **X5** for *Aspergillus niger*, *Aspergillus flavus*, *Penicillium* sp., *Staphylococcus aureus, and Bacillus subtilis* have 19 compounds in the training set and were developed with 3 descriptors. QSAR models **X6** and **X7** for *Escherichia coli* and *Pseudomonas aeruginosa* were developed with a training set with 12 compounds with 2 descriptors. Seven compounds did not have activities against *Escherichia coli* and *Pseudomonas aeruginosa* and are not included in the models. The QSAR equations in Table 3 provided the best leave one out cross-validation criteria (*Q*^2^*_LOO_*) and are free of outliers. The QSAR equations in Table 3 have *R*^2^ values between 0.725 and 0.768 and possess suitable internal validation *Q*^2^*_LOO_* values 0.602 to 0.622 for models **X1**, **X2**, **X3**, **X4**, **X5**, and **X6**. Models **X7** has a *Q*^2^*_LOO_* of 0.510.

The QSAR equations developed for antifungal and antibiotic properties are shown in Table 3, and the plots of observed vs. predicted activities for the models are in Figure 2. Overall, this protocol is not automated and allows for customization. The models were developed using a training set of molecules and validated internally against the training set and externally with the test set. The compounds in the training set include all the compounds with minimum and maximum activities for each of the fungi and bacteria to ensure that external validation of the models was within the QSAR model’s range of development and to avoid extrapolation beyond the training set. The five compounds of the test sets for all seven QSAR models are **1d, 1g**, **1i**, **1l**, and **2i**.

The correlation matrix for the descriptors used in the equations is provided in the Supplementary Material. All cross-correlation values are less than 0.59. Descriptors associated with van der Waals forces, electronegativities, and polarizabilities are important to describe the mycelial growth inhibition of *Aspergillus niger* by the compounds. Steric and electrostatic chargers have been shown by 3-dimensional QSAR to be important to describe antifungal activities against *Aspergillus niger* by 1,4-quinone derivatives [33]. Electronegativities, atomic mass values, and alkyl functional group properties of the compounds are associated with mycelial growth inhibition of *Aspergillus flavus*. Artificial neural networks used complex partial least squares regression analysis to predict antifungal activities for *Aspergillus flavus* for heterocycles [34]. The mycelial growth inhibition of *Penicillium* species by the compounds is described through connectivity properties. Topological and connectivity descriptors were found to be related to antifungal activities of phenolic compounds against *Penicillium expansum* and *Penicillium brevicompactum* [12]. The uniqueness of the QSAR equations for antifungal activity is expected since the antifungal assays used to develop the models do not exhibit uniform biological activities for all species [10]. Furthermore, *Aspergillus flavus, Aspergillus niger* and *Penicillium* species possess unique biology and the production of certain metabolites, including mycotoxins, is species-specific [19].

The minimum inhibitory concentration of *Staphylococcus aureus* by the compounds is explained by electronegativities and the HOMO energy descriptors. Electronic properties, including electronegativities, have been shown to be related to anti-*Staphylococcus aureus* activity by cannabinoids [35]. Inhibition of the growth of Gram-positive *Bacillus subtilis* by the compounds is associated with van der Waals forces, electronegativities, and bandgap energy. Growth inhibition of Gram-negative *Escherichia coli* by the compounds is described by atomic masses and HOMO energy level. Growth inhibition of *Pseudomonas aeruginosa* by the compounds is related to HOMO energy level and polarizabilities. Topological descriptors have been shown to be important components for QSAR models that describe antibacterial growth properties [36]. All QSAR equations for antibacterial activity possessed DFT derived descriptors, although the DFT descriptors are less than 1% of the descriptors considered. This is not unusual; the highest occupied molecular orbital energy level values, *ε_HOMO_*, have been found to be an important descriptor in antimicrobial QSAR studies for other heterocyclic compounds [37,38].

The observed vs. predicted values for the QSAR equations X1, X2, X3, X4, X5, X6, and X7 are given in Figure 2, and the statistic assessment and internal validation of the training sets are provided in Table 4. Table 5 provides the external validation statistics of the test set compounds that were not used in QSAR model development. The correlation coefficient (*R*^2^) and *Q*^2^_LOO_ were previously discussed with the QSAR equations in Table 3. The *s* and *SPRESS* values are related to the residuals of the regression of the QSAR models and the size of the biological activity values. The values of QSAR equations X1, X2, X4, X5, and X7 utilize the log scale to generate suitable equations, which reduces the s and *SPRESS* values. As seen in Figure 2, the calculated values correspond with the observed values, and there are no outliers. The small *p* values indicate strong statistical significance of the models.

Table 5 provides information on the performance of the QSAR models on compounds that were not used to develop the models. The *R*^2^*_ext_* values that measure the correlation coefficient of the test set values using the model developed from the training set are between 0.627 and 0.966. The *F* values are between 5.04 to 87.44 and indicate that predicted activities of the models are associated with the variation of the descriptors. This infers the descriptors are important components of the models and supports the validity of the models. The variability between the test set validation values can be attributed to the differences in activities of the seven different fungi and bacteria investigated and unique activities of triazolothiadiazine compounds that are not fully captured by the models. However, the test set properties indicate the models are suitable for predicting activities for compounds not used to develop the models. The *RMSE* and *MAE* values are excellent, and the scale of these values is related to the magnitude of the activities used to create the models. The mean absolute percentage error (*MAPE*) permits the comparison of the mean absolute error across varying activities and is between 0.5 and 7.0% for the compounds evaluated. Most of the models are at or below 4% mean absolute percentage error, with model X4 as the exception.

The correlation coefficient of regression through the origin (*R*^2^*_ext-RTO_*) is considered an important parameter to characterize the test set values [39,40]. All QSAR equations have *R*^2^*_ext-RTO_* approach 1, which indicates that the descriptors are highly correlated to the activities when regression is through the origin. The *R*^2^*_ext-RTO_* and *R*^2^*_ext-adj_* (not shown) were identical. The *F* values are between 526 to 15,112, which indicates certain models (X1 and X2) were more associated with the descriptors than other models. The *p* values for regression through the origin of QSAR equations were all <0.0001, principally due to the lack of intercept in the regression.

## 4. Conclusions

In conclusion, 2-dimensional topological and quantum chemical descriptors were able to generate QSAR models to describe the antimicrobial activities of triazolothiadiazine compounds against *Aspergillus niger*, *Aspergillus flavus,* and *Penicillium* sp. fungi, as well as, *Staphylococcus aureus, Bacillus subtilis*, *Escherichia coli*, and *Pseudomonas aeruginosa* bacteria. All models include topological descriptors, and the models to describe antibacterial properties include descriptors obtained by DFT methods. These findings will aid in the design of more potent antimicrobial compounds and systems. The method described enabled the rapid development of predictive models to serve as simple tools to screen and evaluate potential antimicrobials based on the triazolothiadiazine substructure. The protocol described provides an efficient method to develop QSAR models against a variety of biological activities.

## Figures and Tables

**Table 1:** Chemical structures and biological activities of triazolothiadiazine compounds investigated in this study.

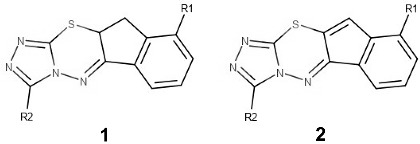										
	Compound	R_1_	R_2_	*A. niger* log(%MGI)	*A. flavus* log(%MGI)	*Penicillium* sp. %MGI	*S. aureus* pIC_50_	*B. subtilis* pIC_50_	*E. coli* DGI	*P. aeruginosa* log(DGI)
1	**1a**	-H	-H	1.71	1.72	51.1	3.25	3.25		
2	**1b**	-H	-CH_3_	1.73	1.71	55.5	2.98	3.28		
3	**1c**	-H	-CH_2_CH_3_	1.77	1.74	57.7	4.20	3.90	18.3	1.26
4	**1d**	-H	-CH_2_CH_2_CH_3_	1.74	1.72	53.3	4.53	4.23	19.3	1.29
5	**1e**	-H	-CH(CH_3_)CH_3_	1.73	1.71	51.1	4.83	4.23	20.6	1.31
6	**1f**	-H	-Ph	1.71	1.69	50.0	3.50	3.20	18.3	1.26
7	**1g**	-CH_3_	-H	1.80	1.82	62.5	4.48	5.08	22.3	1.35
8	**1h**	-CH_3_	-CH_3_	1.83	1.84	67.7	4.51	5.11	20.6	1.31
9	**1i**	-CH_3_	-CH_2_CH_3_	1.81	1.80	65.5	4.53	4.83	21.0	1.32
10	**1j**	-CH_3_	-CH_2_CH_2_CH_3_	1.77	1.74	61.1	4.25	3.95	19.3	1.29
11	**1k**	-CH_3_	-CH(CH_3_)CH_3_	1.80	1.79	65.5	4.85	4.55	21.6	1.33
12	**1l**	-CH_3_	-Ph	1.74	1.77	60.0	4.00	4.00	17.3	1.24
13	**2a**	-H	-H	1.73	1.70	51.1	2.95	3.25		
14	**2b**	-H	-CH_3_	1.70	1.69	52.5	2.97	2.97		
15	**2c**	-H	-CH_2_CH_3_	1.69	1.66	50.0	3.30	3.30		
16	**2d**	-H	-CH_2_CH_2_CH_3_	1.71	1.69	47.7	4.22	4.22	18.6	1.27
17	**2e**	-H	-CH(CH_3_)CH_3_	1.69	1.70	44.4	3.32	3.32		
18	**2f**	-H	-Ph	1.66	1.69	51.1	3.07	3.07		
19	**2g**	-CH_3_	-H	1.85	1.84	66.6	4.18	4.78	20.6	1.31
20	**2h**	-CH_3_	-CH_3_	1.73	1.70	51.1	3.60	3.60	18.6	1.27
21	**2i**	-CH_3_	-CH_2_CH_3_	1.82	1.80	63.3	3.92	3.92	19.3	1.28
22	**2j**	-CH_3_	-CH_2_CH_2_CH_3_	1.84	1.82	65.5	3.34	3.64	15.6	1.19
23	**2k**	-CH_3_	-CH(CH_3_)CH_3_	1.82	1.79	63.3	3.34	3.64	15.0	1.18
24	**2l**	-CH_3_	-Ph	1.74	1.71	48.8	3.69	4.00	15.6	1.19

**Table 2:** Frontier orbital parameters for triazolothiadiazine compounds calculated on geometry optimized structures at the B3LYP/6-311+G** level.

		ε_HOMO_	ε_LUMO_	Δε	μ	*σ*	*η*	*χ*	*ω*
1	**1a**	−6.67	−2.47	4.20	−4.57	0.476	2.10	4.57	4.97
2	**1b**	−6.47	−2.39	4.08	−4.43	0.490	2.04	4.43	4.81
3	**1c**	−6.47	−2.39	4.08	−4.43	0.490	2.04	4.43	4.81
4	**1d**	−6.43	−2.37	4.06	−4.40	0.493	2.03	4.40	4.77
5	**1e**	−6.44	−2.37	4.07	−4.41	0.491	2.04	4.41	4.77
6	**1f**	−6.20	−2.46	3.74	−4.33	0.535	1.87	4.33	5.01
7	**1g**	−6.64	−2.38	4.26	−4.51	0.469	2.13	4.51	4.77
8	**1h**	−6.44	−2.31	4.13	−4.38	0.484	2.07	4.38	4.63
9	**1i**	−6.43	−2.30	4.13	−4.37	0.484	2.07	4.37	4.61
10	**1j**	−6.39	−2.28	4.11	−4.34	0.487	2.08	4.34	4.57
11	**1k**	−6.40	−2.28	4.12	−4.34	0.485	2.06	4.34	4.57
12	**1l**	−6.17	−2.38	3.79	−4.28	0.528	1.90	4.28	4.82
13	**2a**	−6.29	−3.21	3.08	−4.75	0.649	1.54	4.75	7.33
14	**2b**	−6.17	−3.13	3.04	−4.65	0.658	1.52	4.65	7.11
15	**2c**	−6.16	−3.12	3.04	−4.64	0.658	1.52	4.64	7.08
16	**2d**	−6.14	−3.10	3.04	−4.62	0.658	1.52	4.62	7.02
17	**2e**	−6.15	−3.11	3.04	−4.63	0.658	1.52	4.63	7.05
18	**2f**	−6.12	−3.17	2.95	−4.65	0.678	1.48	4.65	7.31
19	**2g**	−6.20	−3.15	3.05	−4.68	0.656	1.53	4.68	7.17
20	**2h**	−6.09	−3.07	3.02	−4.58	0.662	1.51	4.58	6.95
21	**2i**	−6.08	−3.06	3.02	−4.57	0.662	1.51	4.57	6.92
22	**2j**	−6.06	−3.07	2.99	−4.57	0.669	1.50	4.57	6.97
23	**2k**	−6.06	−3.07	2.99	−4.57	0.669	1.50	4.57	6.97
24	**2l**	−6.04	−3.11	2.93	−4.58	0.683	1.45	4.58	7.14

Quantum chemical properties were calculated following published procedures [28,29,30].

**Table 3:** Quantitative structure–activity relationship models of triazolothiadiazine compounds for antifungal and antibiotic activities.

Activity	Eq.	Equation	*R* ^2^	*Q* ^2^ *_LOO_*	*n*
*A. niger*	**X1**	log(%MGI) = − 0.2158(*GTSv6*) − 17.6409(*BELe8*) − 0.2339(*BEHp5*) + 40.537	0.768	0.602	19
*A. flavus*	**X2**	log(%MGI) = 0.2485(*MTam7*) + 2.7052(*BELe3*) + 0.0738(*#RCRR*) − 4.9326	0.725	0.614	19
*Penicillium sp.*	**X3**	%MGI = −11844.7257(*AVC5*) − 0.0083(*MTmp9*) + 0.4168(*CBtpc*) + 843.168	0.735	0.622	19
*S. aureus*	**X4**	pIC_50_ = 1.8950(*GTSe7*) + 4.6320(*BEHe7*) − 4.2409(*ε_HOMO_*) − 41.5777	0.746	0.604	19
*B. subtilis*	**X5**	pIC_50_ = −4.6029(*GTSv3*) + 2.0233(*GTSe7*) + 0.8941(Δε) + 0.5419	0.749	0.613	19
*E. coli*	**X6**	DGI = −13.9498(*BEHm6*) − 6.4231(*ε_HOMO_*) + 85.321	0.763	0.617	12
*P. aeruginosa*	**X7**	log(DGI) = − 1.2342(*GTap2*) − 0.3167(*ε_HOMO_*) + 0.4678	0.754	0.510	12

*GTSv6*, Geary topological structure autocorrelation length-6 weighted by atomic van der Waals volumes; *BELe8*, lowest eigenvalue from Burdex matrix weighted by electronegativities Sanderson scale order-8; *BEHp5*, highest eigenvalue from Burdex matrix weighted by polarizabilities order-5; *MTam7*, Moran topological structure autocorrelation length-7 weighted by atomic masses; *BELe3*, lowest eigenvalue from Burdex matrix weighted by electronegativities Sanderson scale order-3; *#RCRR*, number of group R~CR~R; *AVC5*, average vertex connectivity order-5 index; *MTmp9*, molecular topological multiple path index of order 09; *CBtpc*, ratio of convention bonds with total path counts; *GTSe7*, Geary topological structure autocorrelation length-7 weighted by atomic Sanderson electronegativities; *BEHe7*, highest eigenvalue from Burdex matrix weighted by electronegativities Sanderson-Scale order-7; *ε_HOMO_*, B3LYP/6-311+G** energy of HOMO; *GTSv3*, Geary topological structure autocorrelation length-3 weighted by atomic van der Waals volumes; Δε, B3LYP/6-311+G** bandgap energy; *BEHm6*, highest eigenvalue from Burdex matrix weighted by masses order-6. *GTap2*, Geary topological structure autocorrelation length-2 weighted by atomic polarizabilities.

**Table 4:** Training set parameters of quantitative structure–activity relationship models of triazolothiadiazine compounds for antifungal and antibiotic activities.

	X1 *A. niger*	X2 *A. flavus*	X3 *Penicillium* sp.	X4 *S. aureus*	X5 *B. subtilis*	X6 *E. coli*	X7 *P. aeruginosa*
Training Set							
*R* ^2^	0.768	0.725	0.735	0.746	0.749	0.763	0.754
*Q* ^2^_LOO_	0.602	0.614	0.622	0.604	0.613	0.617	0.510
*R* ^2^ *_adj_*	0.722	0.671	0.681	0.695	0.699	0.711	0.700
*SPRESS*	0.0393	0.0372	4.9296	0.4341	0.4315	1.495	0.0401
*n*	19	19	19	19	19	12	12
*s*	0.03	0.0313	4.130	0.348	0.347	1.176	0.028
*F*	16.56	13.21	13.84	14.68	14.94	14.52	13.84
*p*	0.0001	0.0002	0.0001	0.0001	0.0001	0.0015	0.0018

*Q*^2^, cross-validation; *n*, number of compounds; *s*, standard deviation; *F*, Fisher value; *p*, probability value.

**Table 5:** Test set parameters of quantitative structure–activity relationship models of triazolothiadiazine compounds for antifungal and antibiotic activities.

	X1 *A. niger*	X2 *A. flavus*	X3 *Penicillium* sp.	X4 *S. aureus*	X5 *B. subtilis*	X6 *E. coli*	X7 *P. aeruginosa*
Test Set							
*R_ext_*	0.845	0.955	0.830	0.792	0.983	0.867	0.794
*R* ^2^ *_ext_*	0.713	0.911	0.688	0.627	0.966	0.751	0.631
*R* ^2^ *_ext-adj_*	0.618	0.882	0.584	0.502	0.956	0.668	0.508
*F*	7.47	30.90	6.62	5.04	87.44	9.06	5.13
*RMSE*	0.030	0.018	3.47	0.375	0.152	0.948	0.050
*MAE*	0.025	0.009	2.230	0.300	0.105	0.736	0.043
*MAPE*	1.4	0.5	3.7	7.0	2.4	4.0	3.4
Test Set RTO							
*R* ^2^ *_ext-RTO_*	1	1	0.997	0.992	0.999	0.998	0.998
*F*	15,112	124,471	1456	526	3997	1710	2562
*n*	5	5	5	5	5	5	5

*R_ext_*, correlation coefficient of the test set; *R*^2^*_ext_*, squared correlation coefficient of the test set; *R*^2^*_ext-adj_* adjusted squared correlation coefficient; RMSE, root-mean-square error; *MAE,* mean absolute error; *MAPE, mean absolute percentage error; n*, number of compounds; *s*, standard deviation; *F*, Fisher value; *p*, probability value.

## Data Availability

Not Applicable.

## Supplementary Materials

The following are available online at https://www.mdpi.com/2409-9279/4/1/2/s1, Figure S1: B3LYP/6-311+G** geometry-optimized structures of triazolothiadiazine compounds, Figure S2: Molecular orbitals of triazolothiadiazine compounds optimized using B3LYP/6-311+G** density functional theory, correlation matrix, B3LYP/6-311+G** geometry optimized coordinates.

## References

[1] Quintero F.A., Patel S.J., Muñoz F., Mannan S. (2012). Review of Existing QSAR/QSPR Models Developed for Properties Used in Hazardous Chemicals Classification System. Ind. Eng. Chem. Res..

[2] Muratov E.N., Bajorath J., Sheridan R.P., Tetko I., Filimonov D.A., Poroikov V., Oprea T.I., Baskin I.I., Varnek A., Roitberg A.E. (2020). QSAR without borders. Chem. Soc. Rev..

[3] Shahlaei M. (2013). Descriptor selection methods in quantitative structure–activity relationship studies: A review study. Chem. Rev..

[4] Jenssen H., Fjell C.D., Cherkasov A., Hancock R.E.W. (2008). QSAR modeling and computer-aided design of antimicrobial pep-tides. J. Pept. Sci..

[5] Thabit A.K., Crandon J.L., Nicolau D.P. (2015). Antimicrobial resistance: Impact on clinical and economic outcomes and the need for new antimicrobials. Expert. Opin. Pharmacother..

[6] World Health Organization WHO Fact Sheet, Updated 15 February 2018. https://www.who.int/news-room/fact-sheets/detail/antimicrobial-resistance.

[7] Sharma P.K. (2017). A Review: Antimicrobial Agents Based on Nitrogen and Sulfur Containing Heterocycles. Asian J. Pharm. Clin. Res..

[8] Čačić M., Pavić V., Molnar M., Šarkanj B., Has-Schön E. (2014). Design and synthesis of some new 1, 3, 4-thiadiazines with coumarin moieties and their antioxidative and antifungal activity. Molecules.

[9] Duchowicz P.R., Vitale M.G., Castro E.A., Fernandez M., Caballero J. (2007). QSAR analysis for heterocyclic antifungals. Bioorg. Med. Chem..

[10] Prakash O., Aneja D.K., Hussain K., Lohan P., Ranjan P., Arora S., Sharma C., Aneja K.R. (2011). Synthesis and biological evalu-ation of dihydroindeno and indeno [1,2-e] [1,2,4]triazolo [3,4-b] [1,3,4]thiadiazines as antimicrobial agents. Eur. J. Med. Chem..

[11] Zabka M., Pavela R. (2013). Antifungal efficacy of some natural phenolic compounds against significant pathogenic and toxino-genic filamentous fungi. Chemosphere.

[12] Appell M., Tu Y.-S., Compton D.L., Evans K.O., Wang L.C. (2020). Quantitative structure-activity relationship study for predic-tion of antifungal properties of phenolic compounds. J. Struct. Chem..

[13] Fotso G.W., Ngameni B., Storr T.E., Ngadjui B.T., Mafu S., Stephenson G.R. (2020). Synthesis of novel stilbene–coumarin deriva-tives and antifungal screening of monotes kerstingii-specialized metabolites against fusarium oxysporum. Antibiotics.

[14] Appell M., Bosma W.B. (2015). Assessment of the electronic structure and properties of trichothecene toxins using density func-tional theory. J. Hazard. Mater..

[15] Lima M.A.S., Oliveira M., da Conceição F., de Pimenta A.T.Á., Uchôa P.K.S. (2019). Aspergillus niger: A hundred years of contri-bution to the natural products chemistry. J. Braz. Chem. Soc..

[16] Han X., Jiang H., Li F. (2019). Dynamic ochratoxin A production by strains of Aspergillus niger intended used in food industry of China. Toxins.

[17] Valsecchi I., Stephen-Victor E., Wong S.S.W., Karnam A., Sunde M., Guijarro J.I., de Francisco B.R., Krüger T., Kniemeyer O., Brown G.D. (2020). The role of rodA-conserved cysteine residues in the Aspergillus fumigatus conidial surface organization. J. Fungi.

[18] Xiang F., Zhao Q., Zhao K., Pei H., Tao F. (2020). The Efficacy of Composite Essential Oils against Aflatoxigenic Fungus *Aspergillus flavus* in Maize. Toxins.

[19] Pitt J. (1994). The current role of Aspergillus and Penicillium in human and animal health. J. Med. Vet. Mycol..

[20] Wang X., Shen Y., Thakur K., Han J., Zhang J.-G., Hu F., Wei Z.-J. (2020). Antibacterial activity and mechanism of ginger essen-tial oil against Escherichia coli and Staphylococcus aureus. Molecules.

[21] Witek K., Latacz G., Kaczor A., Czekajewska J., Żesławska E., Chudzik A., Karczewska E., Nitek W., Kieć-Kononowicz K., Handzlik J. (2020). Phenylpiperazine 5,5-Dimethylhydantoin Derivatives as First Synthetic Inhibitors of Msr(A) Efflux Pump in *Staphylococcus epidermidis*. Molecules.

[22] Sivakumar P.M., Kumar T.M., Doble M. (2009). Antifungal Activity, Mechanism and QSAR Studies on Chalcones. Chem. Biol. Drug Des..

[23] Shao Y., Molnar L.F., Jung Y., Kussmann J., Ochsenfeld C., Brown S.T., Gilbert A.T., Slipchenko L.V., Levchenko S.V., O’Neill D.P. (2006). Advances in methods and algorithms in a modern quantum chemistry program package. Phys. Chem. Phys..

[24] Hypercube Inc. (2011). Hyperchem Professional 8.0.10.

[25] O’Boyle N.M., Banck M., James C.A., Morley C., Vandermeersch T., Hutchison G.R. (2011). Open Babel: An open chemical toolbox. J. Cheminform..

[26] Hong H., Xie Q., Ge W., Qian F., Fang H., Shi L., Su Z., Perkins R., Tong W. (2008). Mold (2), molecular descriptors from 2D structures for chemoinformatics and toxicoinformatics. J. Chem. Inf. Model..

[27] de Oliveira D.B., Gaudio A.C. (2000). BuildQSAR: A new computer program for QSAR analysis. Quant. Struct.-Act. Relat..

[28] Koopmans T. (1934). Über die Zuordnung von Wellenfunktionen und Eigenwerten zu den Einzelnen Elektronen Eines Atoms. Physica.

[29] Parr R.G., Szentpály L.V., Liu S. (1999). Electrophilicity Index. J. Am. Chem. Soc..

[30] Domingo L.R., Ríos-Gutiérrez M., Pérez P. (2016). Applications of the Conceptual Density Functional Theory Indices to Organic Chemistry Reactivity. Molecules.

[31] Martin T., Harten P., Young D.M., Muratov E.N., Golbraikh A., Zhu H., Tropsha A. (2012). Does Rational Selection of Training and Test Sets Improve the Outcome of QSAR Modeling?. J. Chem. Inf. Model..

[32] Rogers D., Hopfinger A.J. (1994). Application of Genetic Function Approximation to Quantitative Structure-Activity Relationships and Quantitative Structure-Property Relationships. J. Chem. Inf. Model..

[33] Choi S.-Y., Shin J.H., Ryu C.K., Nam K.-Y., No K.T., Choo H.-Y.P. (2006). The development of 3D-QSAR study and recursive partitioning of heterocyclic quinone derivatives with antifungal activity. Bioorg. Med. Chem..

[34] Karadžić M.Z., Kovačević S.Z., Jevrić L.R., Podunavac-Kuzmanović S.O. (2017). Chemometric and QSAR analysis of some thiadi-azines as potential antifungal agents. Acta Period. Technol..

[35] Cortes E., Mora J., Marquez E. (2020). Modelling the anti-methicillin-resistant Staphylococcus aureus (MRSA) activity of canna-binoids: A QSAR and docking study. Crystals.

[36] Bouarab-Chibane L., Forquet V., Lantéri P., Clément Y., Léonard-Akkari L., Oulahal N., Degraeve P., Bordes C. (2019). Anti-bacterial properties of polyphenols: Characterization and QSAR (quantitative structure–activity relationship) models. Front. Microbiol..

[37] Mor S., Pahal P., Narasimhan B. (2012). Synthesis, characterization, antimicrobial activities and QSAR studies of some 10a-phenylbenzo[b]indeno[1,2-e][1,4]thiazin-11(10aH)-ones. Eur. J. Med. Chem..

[38] Sabet R., Fassihi A. (2008). QSAR Study of Antimicrobial 3-Hydroxypyridine-4-one and 3-Hydroxypyran-4-one Derivatives Using Different Chemometric Tools. Int. J. Mol. Sci..

[39] Tropsha A. (2010). Best Practices for QSAR Model Development, Validation, and Exploitation. Mol. Inform..

[40] Alexander D.L.J., Tropsha A., Winkler D.A. (2015). Beware of R2: Simple, Unambiguous Assessment of the Prediction Accuracy of QSAR and QSPR Models. J. Chem. Inf. Model..

