# Supplementary material for: Predictive Quantitative Structure–Activity Relationship Modeling of the Antifungal and Antibiotic Properties of Triazolothiadiazine Compounds
Source: Methods Protoc. 2020 Dec 27;4(1):2. doi: 10.3390/mps4010002 (PMC7838911; doi:10.3390/mps4010002)
Supplement: Supplementary file 1 [file mps-04-00002-s001.pdf]

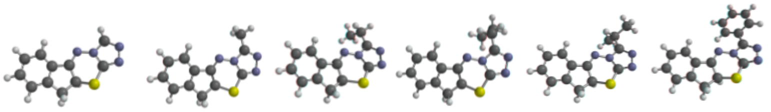

1a 1b 1c 1d 1e 1f

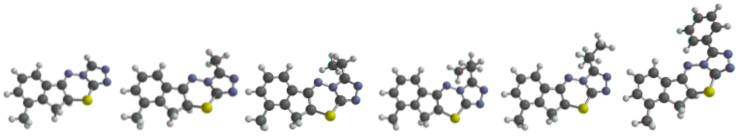

1g 1h 1i 1j 1k 1l

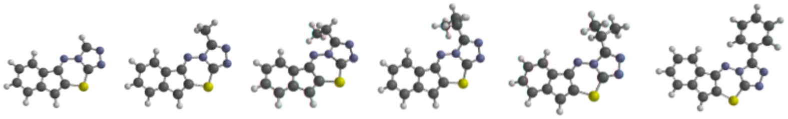

2a 2b 2c 2d 2e 2f

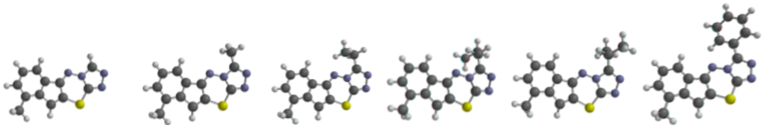

2g 2h 2i 2j 2k 2l

Figure S1. B3LYP/6-311+G** geometry-optimized structures of triazolothiadiazine compounds.

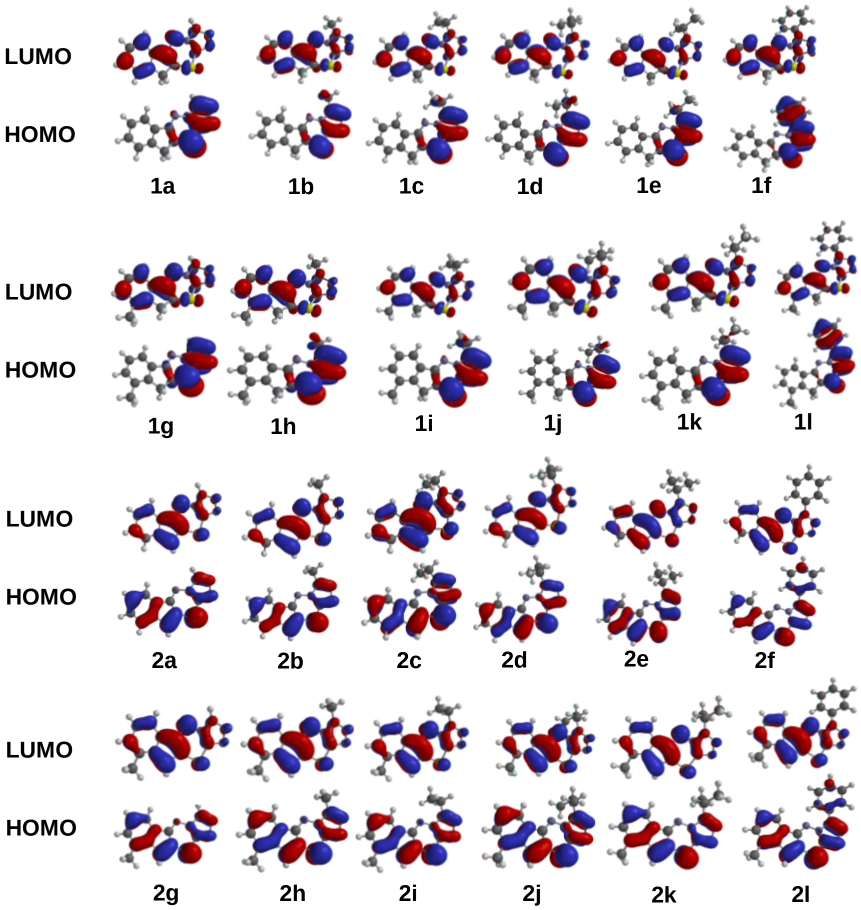

Figure S2. Molecular orbitals of triazolothiadiazine compounds optimized using B3LYP/6-311+G** density functional theory.

Correlation Matrix

X1 ***Aspergillus niger***

	GTSv6	*BELe8*	*BEHp5*
GTSv6	1	0.257	0.136
BELe8	0.257	1	0.370
BEHp5	0.136	0.370	1

X2 ***Aspergillus flavus***

	MTam75	*BELe3*	*#RCRR*
MTam7	1	0.427	0.444
BELe3	0.427	1	0.589
#RCRR	0.444	0.589	1

X3 ***Penicillium sp.***

	AVC5	*MTmp9*	*CBtpc*
AVC5	1	0.534	0.269
MTmp9	0.534	1	0.559
CBtpc	0.269	0.559	1

X4 ***Staphylococcus aureus***

	GTSe7	*BEHe7*	ε_{HOMO}
GTSe7	1	0.031	0.505
BEHe7	0.031	1	0.534
ε_{HOMO}	0.505	0.534	1

X5 ***Bacillus subtilis***

	GTSv3	*GTSe7*	$\Delta\varepsilon$
GTSv3	1	0.456	0.028
GTSe7	0.456	1	0.375
$\Delta\varepsilon$	0.028	0.375	1

X6 ***Escherichia coli***

	BEHm6	ε_{HOMO}
BEHm6	1	0.453
ε_{HOMO}	0.453	1

X7 ***Pseudomonas aeruginosa***

	GTap2	ε_{HOMO}
GTap2	1	0.556
ε_{HOMO}	0.556	1

B3LYP/6-311+G** geometry optimized coordinates

1a

C	2.223200	0.639276	-0.049520
C	2.846078	1.881271	-0.087008
C	2.085619	3.057588	-0.126476
C	0.691694	3.011325	-0.125413
C	0.061581	1.771033	-0.087374
C	0.826489	0.596551	-0.056113
C	-0.065552	-0.559054	-0.044093
C	-1.485958	-0.062071	-0.266463
C	-1.420706	1.465587	-0.036795
N	0.317338	-1.774882	0.110337
S	-2.676032	-0.929353	0.843117
C	-2.032889	-2.525537	0.497269
N	-0.703358	-2.701974	0.165408
C	-0.554918	-4.065098	0.082750
N	-1.689378	-4.660984	0.318563
N	-2.636709	-3.679459	0.588136
H	2.797762	-0.278811	-0.021214
H	3.928066	1.943180	-0.088512
H	2.588669	4.017506	-0.157652
H	0.113357	3.928600	-0.151502
H	-1.774770	-0.286318	-1.297791
H	-1.827384	1.727456	0.946127
H	-1.995108	2.016259	-0.784518
H	0.382909	-4.532088	-0.171263

1b

C	1.190535	-2.118646	-0.125792
C	2.444952	-2.750891	-0.095187
C	3.620838	-2.000617	-0.042793
C	3.586648	-0.594356	-0.020029
C	2.352879	0.036254	-0.053270
C	1.154853	-0.730537	-0.108436
C	0.004631	0.188963	-0.111735
C	0.549648	1.596222	-0.337563
C	2.056470	1.515856	-0.031994
N	-1.229187	-0.171616	0.114971
S	-0.292171	2.780931	0.761432
C	-1.906871	2.207858	0.533240
N	-2.158752	0.825444	0.262733
C	-3.580500	0.714140	0.201881
N	-4.085820	1.955250	0.443582
N	-3.087240	2.847775	0.640798
H	0.268458	-2.699635	-0.160427
H	2.495426	-3.839258	-0.110286
H	4.585772	-2.508896	-0.017925
H	4.511157	-0.023263	0.024056

H	0.377561	1.894880	-1.401177
H	2.316675	1.947027	0.957996
H	2.656673	2.074207	-0.774776
C	-4.307216	-0.531417	-0.078564
H	-3.932420	-1.369794	0.533792
H	-4.206927	-0.829526	-1.135806
H	-5.386072	-0.416356	0.131280

1c

C	2.180052	-1.334810	0.190146
C	3.561189	-1.485441	0.225152
C	4.394214	-0.370904	0.388770
C	3.859327	0.909692	0.526273
C	2.476921	1.067460	0.493978
C	1.648056	-0.049071	0.319374
C	0.253905	0.384358	0.297807
C	0.227713	1.904148	0.296733
C	1.666025	2.335459	0.665251
N	-0.757235	-0.407445	0.268853
S	-1.059884	2.534280	1.456869
C	-2.307691	1.454993	0.854100
N	-1.986033	0.213150	0.335473
C	-3.205923	-0.388430	0.085287
N	-4.167820	0.436872	0.405538
N	-3.599584	1.607917	0.898988
H	1.523503	-2.187107	0.063022
H	3.999613	-2.471434	0.123471
H	5.469680	-0.507173	0.411917
H	4.513662	1.764549	0.658907
H	-0.036766	2.249833	-0.706992
H	1.715087	2.678882	1.704541
H	2.019115	3.154672	0.035499
C	-3.360183	-1.746204	-0.509647
H	-2.715546	-2.446530	0.030569
H	-4.395186	-2.044210	-0.333497
C	-3.038390	-1.800338	-2.013069
H	-3.184403	-2.814228	-2.393797
H	-3.691323	-1.128266	-2.573909
H	-2.002095	-1.514677	-2.205607

1d

C	2.506194	-1.164213	0.734948
C	3.882442	-1.281510	0.888671
C	4.682531	-0.139919	1.029067
C	4.118771	1.135675	1.021738
C	2.741119	1.259992	0.870065
C	1.945199	0.115818	0.722032
C	0.548782	0.516349	0.569934
C	0.499807	2.030707	0.445489

C	1.901061	2.520115	0.877784
N	-0.444261	-0.296880	0.541292
S	-0.871884	2.724072	1.462866
C	-2.059915	1.568112	0.882173
N	-1.686705	0.296122	0.483076
C	-2.881845	-0.351962	0.219966
N	-3.873562	0.474944	0.424607
N	-3.355588	1.694761	0.846353
H	1.875410	-2.038003	0.624111
H	4.342922	-2.262671	0.898228
H	5.754593	-0.250749	1.147261
H	4.746974	2.012374	1.136799
H	0.302487	2.291065	-0.598698
H	1.870372	2.948559	1.885646
H	2.286683	3.292002	0.208427
C	-2.987910	-1.759252	-0.269905
H	-2.029285	-2.258825	-0.107367
H	-3.732962	-2.267214	0.348681
C	-3.401439	-1.869303	-1.752219
H	-3.551087	-2.930166	-1.980284
H	-4.371220	-1.379866	-1.881037
C	-2.384549	-1.273241	-2.728339
H	-2.719568	-1.395466	-3.761848
H	-2.245163	-0.201978	-2.555454
H	-1.408405	-1.759452	-2.634064

1e

C	2.712338	-0.890432	-0.396015
C	4.079075	-0.930686	-0.645005
C	4.843158	0.243172	-0.611495
C	4.254022	1.474163	-0.324445
C	2.886386	1.521190	-0.071737
C	2.123862	0.345841	-0.116108
C	0.726549	0.666878	0.161277
C	0.588598	2.179788	0.214227
C	2.035794	2.715898	0.306770
N	-0.206325	-0.202381	0.312775
S	-0.483640	2.689367	1.624715
C	-1.739065	1.516474	1.262727
N	-1.439027	0.313237	0.647306
C	-2.632865	-0.389170	0.651374
N	-3.562701	0.347757	1.198627
N	-2.998105	1.558393	1.590762
H	2.109250	-1.789928	-0.420648
H	4.559901	-1.876202	-0.868765
H	5.907715	0.192408	-0.810862
H	0.106371	2.519146	-0.707296
H	2.266823	3.033718	1.329522
H	2.195805	3.577020	-0.345046

C	-2.789503	-1.753788	0.050274
C	-3.995148	-2.484866	0.656596
H	-1.876913	-2.310904	0.292726
C	-2.889018	-1.668795	-1.487065
H	-2.990875	-2.671132	-1.912004
H	-3.759501	-1.080752	-1.787529
H	-4.080449	-3.482497	0.217561
H	-3.890851	-2.589732	1.738663
H	-4.920861	-1.939108	0.462604
H	-1.997093	-1.209738	-1.918838
H	4.856291	2.375661	-0.295650

1f

C	2.772296	-0.654044	0.396096
C	4.154586	-0.774805	0.474657
C	4.962342	0.364370	0.586518
C	4.400675	1.640533	0.628498
C	3.017003	1.768541	0.552224
C	2.213474	0.626873	0.428020
C	0.812655	1.030312	0.347186
C	0.752532	2.546218	0.281033
C	2.180818	3.029302	0.623206
N	-0.181109	0.216821	0.332935
S	-0.538346	3.167600	1.440895
C	-1.763433	2.047596	0.873639
N	-1.426649	0.812458	0.345421
C	-2.648020	0.185404	0.126314
N	-3.613251	1.006440	0.476154
N	-3.058740	2.176327	0.953285
H	2.134664	-1.525976	0.311388
H	4.613095	-1.756472	0.448499
H	6.039200	0.251444	0.645015
H	5.036035	2.514712	0.723069
H	0.462141	2.850961	-0.728563
H	2.219713	3.454362	1.632265
H	2.522537	3.803851	-0.066862
C	-2.863515	-1.147799	-0.443136
C	-1.886884	-1.837528	-1.174588
C	-4.125250	-1.739976	-0.264666
C	-2.167928	-3.092361	-1.710311
C	-3.419713	-3.674409	-1.527585
C	-4.397972	-2.990518	-0.803831
H	-0.912226	-1.396603	-1.325823
H	-4.878616	-1.202172	0.296421
H	-1.403349	-3.614531	-2.275540
H	-3.633183	-4.651566	-1.946125
H	-5.375581	-3.435367	-0.655707

1g

C	0.076368	-2.274188	0.008130
C	1.312574	-2.906174	0.006423
C	2.496479	-2.158552	-0.030379
C	2.490911	-0.760464	-0.065277
C	1.241587	-0.129805	-0.068857
C	0.057402	-0.878108	-0.035945
C	-1.086902	0.028077	-0.052852
C	-0.572092	1.437333	-0.297991
C	0.953461	1.357059	-0.065023
N	-2.308801	-0.335729	0.102092
S	-1.424206	2.656394	0.792216
C	-3.028815	2.029468	0.453862
N	-3.221328	0.698705	0.136192
C	-4.586490	0.565870	0.057492
N	-5.168126	1.710201	0.280809
N	-4.175018	2.648993	0.537895
H	-0.848901	-2.836165	0.038779
H	1.367850	-3.988375	0.034404
H	3.448598	-2.679301	-0.027966
C	3.772423	0.033797	-0.086001
H	-0.792002	1.712568	-1.333863
H	1.224316	1.796675	0.902212
H	1.509523	1.902333	-0.831174
H	-5.065755	-0.368821	-0.184794
H	3.840642	0.664529	-0.978110
H	4.643582	-0.622239	-0.074603
H	3.842723	0.695920	0.782330

1h

C	1.355173	-1.788415	-0.137144
C	2.732673	-1.948242	-0.071490
C	3.571417	-0.842061	0.114499
C	3.068688	0.456408	0.245248
C	1.679640	0.611109	0.173514
C	0.841357	-0.494933	-0.018304
C	-0.547306	-0.046839	-0.061637
C	-0.559107	1.472032	-0.061420
C	0.879603	1.889143	0.319177
N	-1.566977	-0.826980	-0.103106
S	-1.846280	2.114877	1.092312
C	-3.102943	1.047028	0.487249
N	-2.788810	-0.191386	-0.047237
C	-4.013889	-0.784674	-0.291959
N	-4.970088	0.041894	0.039708
N	-4.393923	1.206642	0.539099
H	0.691631	-2.632764	-0.277719
H	3.168052	-2.936647	-0.163958
H	4.644579	-0.996412	0.163380

C	3.981954	1.637409	0.459556
H	-0.813753	1.822135	-1.066098
H	0.920362	2.251902	1.352966
H	1.246877	2.697541	-0.317210
C	-4.175157	-2.143626	-0.871162
H	-3.636178	-2.888328	-0.278696
H	-5.237178	-2.387842	-0.880837
H	-3.790001	-2.191200	-1.894046
H	3.883417	2.371890	-0.346901
H	3.749192	2.154521	1.396652
H	5.026973	1.325817	0.505564

1i

C	1.700220	0.170219	1.582427
C	3.078347	0.239740	1.735782
C	3.912642	0.403671	0.622772
C	3.404684	0.509350	-0.676244
C	2.014941	0.437270	-0.823858
C	1.181270	0.264857	0.288927
C	-0.209176	0.211256	-0.152825
C	-0.227778	0.189890	-1.671309
C	1.209273	0.562516	-2.100638
N	-1.225125	0.177571	0.632496
S	-1.519633	1.334007	-2.321518
C	-2.769798	0.733221	-1.243803
N	-2.450566	0.220400	0.001473
C	-3.672304	-0.031285	0.599570
N	-4.631998	0.281971	-0.231328
N	-4.061664	0.771324	-1.402970
H	1.039832	0.044464	2.431678
H	3.517555	0.166060	2.724097
H	4.986467	0.454701	0.771679
C	4.313004	0.695996	-1.865342
H	-0.485791	-0.819134	-2.006636
H	1.250193	1.590208	-2.480522
H	1.572397	-0.087384	-2.900385
C	-3.829222	-0.621724	1.959325
H	-3.199837	-0.067020	2.662189
H	-4.869954	-0.460963	2.247418
C	-3.483226	-2.119417	2.024183
H	-3.641965	-2.499431	3.036314
H	-4.113885	-2.693769	1.341573
H	-2.438430	-2.295494	1.758738
H	4.179063	-0.105071	-2.599677
H	4.108897	1.640862	-2.380302
H	5.361569	0.701137	-1.563286

1j

C	2.052296	-1.395027	0.412697
C	3.435730	-1.532261	0.612792
C	4.236001	-0.420795	0.873833
C	3.688995	0.879020	0.945030
C	2.317573	1.020015	0.740025
C	1.509868	-0.118920	0.476247
C	0.108739	0.318157	0.329861
C	0.116315	1.839942	0.234664
C	1.492586	2.283697	0.763995
N	-0.919681	-0.484260	0.360583
S	-1.238758	2.539585	1.231488
C	-2.481332	1.439292	0.749502
N	-2.164995	0.088025	0.400462
C	-3.422270	-0.527738	0.127243
N	-4.379262	0.418231	0.334018
N	-3.818805	1.593469	0.703811
H	1.421978	-2.261687	0.216822
H	3.886319	-2.523762	0.565324
H	5.306840	-0.555654	1.028223
C	4.554317	2.060173	1.228565
H	-0.015035	2.146933	-0.832019
H	1.443839	2.692014	1.795170
H	1.934812	3.081055	0.137826
C	-3.604277	-1.933632	-0.304590
H	-2.706636	-2.539344	-0.047042
H	-4.450418	-2.377949	0.269491
C	-3.897146	-2.031170	-1.811617
H	-4.236315	-3.060632	-2.042833
H	-4.748291	-1.365267	-2.067943
C	-2.684921	-1.679111	-2.666982
H	-2.900623	-1.815032	-3.733091
H	-2.383155	-0.634349	-2.525427
H	-1.817958	-2.303760	-2.421940
H	4.595798	2.740754	0.365039
H	5.589968	1.780695	1.465875
H	4.177902	2.639291	2.084897

1k

C	2.285448	-1.185702	-0.327502
C	3.662618	-1.240726	-0.492133
C	4.441333	-0.082691	-0.378141
C	3.878259	1.166336	-0.096552
C	2.489405	1.215287	0.066798
C	1.709861	0.057242	-0.052906
C	0.305601	0.395811	0.158478
C	0.187934	1.906465	0.262283
C	1.637629	2.417886	0.419757
N	-0.648602	-0.460417	0.231804

S	-0.921634	2.373357	1.659307
C	-2.185420	1.244211	1.198336
N	-1.886198	0.067657	0.532745
C	-3.094432	-0.605296	0.454191
N	-4.029275	0.123582	1.004185
N	-3.455606	1.298619	1.480676
H	1.668379	-2.071960	-0.408932
H	4.145149	-2.187076	-0.708406
H	5.516173	-0.156305	-0.508366
H	-0.263398	2.288829	-0.658023
H	1.831844	2.732653	1.451706
H	1.833088	3.282163	-0.219328
C	-3.274359	-1.924697	-0.237805
C	-4.156948	-2.863866	0.599759
H	-2.278123	-2.366739	-0.338150
C	-3.854413	-1.717432	-1.652223
H	-3.945021	-2.678074	-2.167144
H	-4.845327	-1.260485	-1.593550
H	-4.299306	-3.811171	0.072524
H	-3.699621	-3.075844	1.569429
H	-5.136019	-2.415544	0.780169
H	-3.214620	-1.068454	-2.256461
C	4.731477	2.404303	0.017804
H	5.792457	2.150920	0.032535
H	4.562768	3.081091	-0.826583
H	4.508899	2.966067	0.929719

1l

C	2.300325	-0.962758	0.417202
C	3.677784	-1.091167	0.533379
C	4.494966	0.042386	0.630775
C	3.969599	1.338635	0.620961
C	2.580384	1.460955	0.506015
C	1.764130	0.327470	0.398499
C	0.370186	0.745636	0.283374
C	0.326934	2.259342	0.186079
C	1.759769	2.734053	0.516076
N	-0.634560	-0.054657	0.272048
S	-0.955992	2.914297	1.337263
C	-2.195130	1.804758	0.780258
N	-1.871276	0.561737	0.261172
C	-3.099314	-0.048699	0.035563
N	-4.056189	0.786241	0.374542
N	-3.488808	1.951867	0.849037
H	1.653157	-1.828424	0.344543
H	4.130078	-2.076167	0.549672
H	5.568712	-0.088039	0.720564
C	4.858600	2.552230	0.725552
H	0.035289	2.548357	-0.827575

H	1.800210	3.209400	1.502772
H	2.114309	3.473410	-0.206482
C	-3.328949	-1.377848	-0.538255
C	-2.356591	-2.075938	-1.267480
C	-4.601111	-1.952396	-0.375038
C	-2.651498	-3.321111	-1.817648
C	-3.915002	-3.884386	-1.652159
C	-4.888654	-3.192903	-0.929416
H	-1.374254	-1.647085	-1.406975
H	-5.351917	-1.408917	0.186119
H	-1.889977	-3.848734	-2.381813
H	-4.141768	-4.852742	-2.083908
H	-5.875077	-3.622900	-0.797988
H	4.775377	3.183273	-0.166219
H	5.906782	2.266288	0.835037
H	4.589476	3.174537	1.584457

2a

C	-0.964084	0.024283	2.017983
C	-0.469775	0.039808	3.339347
C	0.899369	0.041310	3.593729
C	1.840500	0.026942	2.542595
C	1.368014	0.011358	1.243148
C	-0.045705	0.010476	0.986238
C	-0.217224	-0.006647	-0.485296
C	1.171838	-0.017034	-1.049216
C	2.085017	-0.006345	-0.050469
N	-1.355163	-0.010399	-1.123815
S	1.459619	-0.041130	-2.753874
C	-0.165573	-0.039554	-3.340960
N	-1.310205	-0.025816	-2.487828
C	-2.425286	-0.031253	-3.373583
N	-1.941150	-0.046903	-4.636058
N	-0.588107	-0.051916	-4.624843
H	-2.036360	0.023329	1.821296
H	-1.177178	0.050763	4.168887
H	1.258770	0.053764	4.623783
H	2.906893	0.028235	2.758533
H	-3.454652	-0.023767	-3.047107
H	3.160444	-0.009501	-0.122489

2b

C	0.448012	-0.023516	2.307895
C	-0.021546	-0.041314	3.626930
C	-1.391630	-0.046077	3.891337
C	-2.332037	-0.033373	2.853003
C	-1.871620	-0.015198	1.542281
C	-0.482502	-0.010409	1.282177
C	-0.321343	0.008654	-0.178403

C	-1.689684	0.014718	-0.751285
C	-2.590037	0.000764	0.262552
N	0.809822	0.015716	-0.791533
S	-1.992031	0.045431	-2.482975
C	-0.320815	0.035490	-3.019641
N	0.761385	0.031070	-2.153923
C	1.871205	0.028988	-2.986394
N	1.477532	0.033036	-4.229503
N	0.082806	0.038494	-4.253974
H	1.510215	-0.019896	2.093043
H	0.684713	-0.051339	4.448738
H	-1.735861	-0.059910	4.919475
H	-3.393603	-0.037946	3.073566
C	3.272256	0.022513	-2.493564
H	3.471512	-0.858312	-1.877814
H	3.936970	0.014371	-3.356578
H	3.483027	0.906751	-1.886621
H	-3.666746	0.001294	0.161214

2c

C	-0.009272	-2.500885	0.263826
C	0.470217	-3.815959	0.290429
C	1.842521	-4.069961	0.283615
C	2.774809	-3.024716	0.250507
C	2.303916	-1.717681	0.223706
C	0.913075	-1.468032	0.230709
C	0.740912	-0.008747	0.195626
C	2.104707	0.574217	0.170499
C	3.012564	-0.432970	0.186308
N	-0.395051	0.595356	0.188232
S	2.393452	2.307842	0.126943
C	0.716855	2.830862	0.132382
N	-0.358670	1.957911	0.151945
C	-1.475483	2.782952	0.152476
N	-1.088928	4.027990	0.131170
N	0.305431	4.062219	0.118452
H	-1.073049	-2.294556	0.269354
H	-0.229369	-4.642964	0.316142
H	2.194453	-5.095285	0.304308
H	3.837999	-3.236945	0.245849
C	-2.880490	2.284430	0.147046
H	-2.987752	1.504024	0.907380
H	-3.505735	3.128101	0.448651
C	-3.334309	1.745477	-1.221160
H	-4.382315	1.437746	-1.176944
H	-3.237174	2.514233	-1.990456
H	-2.741772	0.878886	-1.520733
H	4.088457	-0.323544	0.173740

2d

C	-1.035800	-2.938500	0.129200
C	-0.555600	-4.259000	0.174500
C	0.831000	-4.528700	0.190300
C	1.775300	-3.486200	0.162000
C	1.280700	-2.183500	0.116200
C	-0.077100	-1.929100	0.099400
C	-0.239700	-0.491700	0.068100
C	1.105500	0.058400	0.068100
C	2.033900	-0.921500	0.092800
N	-1.367600	0.206000	0.068100
S	1.380200	1.814900	0.078200
C	-0.292900	2.439100	0.091800
N	-1.367600	1.667800	0.068100
C	-2.464600	2.408900	0.098800
N	-2.094700	3.680900	0.136900
N	-0.645000	3.703800	0.134200
H	-2.093000	-2.718400	0.118100
H	-1.257300	-5.079600	0.196900
H	1.172000	-5.552800	0.226000
H	2.836600	-3.686200	0.175200
C	-3.894100	1.892300	0.091700
H	-4.055400	1.257500	0.963000
H	-4.585000	2.734800	0.123000
C	-4.138400	1.075500	-1.190700
H	-5.163500	0.705000	-1.195800
H	-3.977100	1.710200	-2.062000
C	-3.162200	-0.114800	-1.235000
H	-3.335100	-0.693000	-2.142700
H	-2.137100	0.255600	-1.229900
H	-3.323500	-0.749600	-0.363700
H	3.107400	-0.802800	0.094100

2e

C	0.373519	-2.854064	-0.258058
C	0.846915	-4.171492	-0.291756
C	2.217319	-4.432843	-0.275568
C	3.154452	-3.392681	-0.227273
C	2.690420	-2.083214	-0.195761
C	1.300575	-1.826234	-0.211203
C	1.135151	-0.366500	-0.159912
C	2.501889	0.208694	-0.122780
C	3.405127	-0.802587	-0.143032
N	0.001921	0.243039	-0.141313
S	2.797615	1.941165	-0.090861
C	1.124208	2.470513	-0.042722
N	0.043139	1.603864	-0.074459
C	-1.070909	2.435086	-0.040384
N	-0.674847	3.676349	0.007118

N	0.719455	3.703088	0.005925
H	-0.689244	-2.642167	-0.269686
H	0.143149	-4.994612	-0.330394
H	2.564558	-5.460023	-0.300909
C	-2.481460	1.925299	-0.020195
C	-2.848335	1.403061	1.384654
H	-2.522809	1.077951	-0.713465
C	-3.460870	3.005182	-0.498240
H	-4.477102	2.602629	-0.502570
H	-3.434551	3.877101	0.156819
H	-3.866711	1.005088	1.383687
H	-2.174252	0.604836	1.703485
H	-2.799015	2.212210	2.118602
H	-3.217314	3.341010	-1.508259
H	4.216541	-3.610947	-0.214651
H	4.481477	-0.698802	-0.126840

2f

C	-0.875530	-0.160366	2.760200
C	-1.384429	-0.234997	4.062391
C	-2.761567	-0.225000	4.287027
C	-3.670274	-0.142150	3.223981
C	-3.170339	-0.066067	1.930218
C	-1.774313	-0.074521	1.710067
C	-1.570606	0.017432	0.256781
C	-2.920874	0.071189	-0.352296
C	-3.851043	0.024843	0.632825
N	-0.420111	0.046945	-0.318118
S	-3.162914	0.187901	-2.087602
C	-1.475977	0.167565	-2.568251
N	-0.412512	0.137308	-1.680145
C	0.718772	0.142909	-2.496540
N	0.332539	0.177453	-3.748813
N	-1.049677	0.192079	-3.796305
H	0.192509	-0.172390	2.577702
H	-0.703258	-0.301672	4.902382
H	-3.135956	-0.283544	5.302814
H	-4.737904	-0.138172	3.412507
C	2.121076	0.096786	-2.066484
C	2.558527	0.536011	-0.808899
C	3.066381	-0.376488	-2.991592
C	3.912615	0.492348	-0.485538
C	4.843817	0.015480	-1.404772
C	4.414772	-0.415003	-2.660603
H	1.845669	0.906764	-0.088576
H	2.726037	-0.706163	-3.964495
H	4.239641	0.838190	0.489280
H	5.896271	-0.018506	-1.146618
H	5.132819	-0.785632	-3.383407

H	-4.924160	0.049468	0.500880

2g

C	-1.420495	0.012286	1.623355
C	-0.946917	0.029887	2.941245
C	0.419954	0.036782	3.200713
C	1.384692	0.027126	2.174196
C	0.906601	0.008616	0.864396
C	-0.485254	0.001161	0.605182
C	-0.653894	-0.017669	-0.854491
C	0.710203	-0.022568	-1.433447
C	1.614861	-0.006229	-0.424419
N	-1.789344	-0.024045	-1.460315
S	1.007886	-0.056966	-3.168380
C	-0.665022	-0.047112	-3.695973
N	-1.740191	-0.037778	-2.826206
C	-2.840570	-0.037503	-3.656524
N	-2.468265	-0.049361	-4.902833
N	-1.076904	-0.053066	-4.932581
H	-2.481868	0.006740	1.406140
H	-1.649419	0.037536	3.766432
H	0.759676	0.049788	4.231939
C	2.854530	0.040633	2.516202
H	-3.848955	-0.028154	-3.275857
H	3.113735	-0.805173	3.160022
H	3.485183	-0.013833	1.628751
H	3.120828	0.954292	3.056467
H	2.688949	-0.005390	-0.544012

2h

C	-0.911290	0.026872	1.942426
C	-0.438651	0.035969	3.260722
C	0.928578	0.033551	3.521384
C	1.893149	0.023338	2.495536
C	1.415981	0.015914	1.185155
C	0.024211	0.016832	0.924650
C	-0.142770	0.002851	-0.535336
C	1.220889	-0.002420	-1.113149
C	2.125128	0.005023	-0.103067
N	-1.277350	-0.007438	-1.142796
S	1.517787	-0.009178	-2.846834
C	-0.155793	-0.028510	-3.376294
N	-1.234033	-0.026306	-2.506055
C	-2.347248	-0.041239	-3.333032
N	-1.959396	-0.050241	-4.578135
N	-0.565054	-0.043893	-4.608855
H	-1.972616	0.027486	1.724575
H	-1.141608	0.043914	4.085613
H	1.267522	0.039199	4.552954

C	3.363070	0.028231	2.836219
C	-3.745936	-0.045783	-2.833291
H	-3.955517	0.844383	-2.234414
H	-4.415223	-0.064937	-3.692657
H	-3.939934	-0.920358	-2.207090
H	3.610489	-0.794459	3.513968
H	3.991833	-0.072508	1.950860
H	3.644450	0.959005	3.338572
H	3.199333	0.004701	-0.221625

2i

C	-0.475930	-2.150602	0.240501
C	0.004842	-3.465893	0.261024
C	1.372985	-3.718309	0.251127
C	2.331318	-2.686495	0.221915
C	1.846269	-1.379009	0.201709
C	0.452934	-1.126516	0.210237
C	0.277466	0.332420	0.178948
C	1.638277	0.917127	0.156310
C	2.548210	-0.087780	0.169526
N	-0.860446	0.933473	0.170384
S	1.924780	2.652311	0.120668
C	0.247632	3.172160	0.117740
N	-0.826774	2.297053	0.133276
C	-1.945635	3.119968	0.127177
N	-1.561332	4.366629	0.111164
N	-0.167644	4.403632	0.102926
H	-1.538519	-1.939748	0.247848
H	-0.693095	-4.294707	0.283760
H	1.718238	-4.747648	0.266387
C	3.803897	-3.017793	0.217331
C	-3.349647	2.619430	0.116601
H	-3.464320	1.856878	0.892601
H	-3.981051	3.467586	0.392082
C	-3.786934	2.051296	-1.244626
H	-4.834101	1.743745	-1.202225
H	-3.684786	2.804042	-2.030674
H	-3.187892	1.180102	-1.522765
H	4.055992	-3.665099	-0.627810
H	4.425730	-2.124797	0.148280
H	4.087907	-3.549766	1.130374
H	3.621632	0.036312	0.158208

2j

C	-0.008984	-2.371654	0.790710
C	0.525309	-3.657776	0.916103
C	1.900939	-3.868657	0.816636
C	2.807364	-2.819642	0.590520
C	2.265795	-1.538025	0.468207

C	0.872740	-1.327017	0.568850
C	0.641982	0.113538	0.386793
C	1.974312	0.729163	0.181091
C	2.918079	-0.243468	0.229185
N	-0.514665	0.677825	0.399901
S	2.192141	2.457077	-0.055578
C	0.498195	2.918501	-0.024941
N	-0.538126	2.026006	0.198414
C	-1.685905	2.808509	0.151491
N	-1.349165	4.048218	-0.073511
N	0.036992	4.121686	-0.187711
H	-1.075382	-2.195954	0.865758
H	-0.133236	-4.500744	1.090950
H	2.287491	-4.877949	0.917065
C	4.289327	-3.072402	0.478652
C	-3.072633	2.278661	0.325108
H	-3.010452	1.230214	0.626273
H	-3.538681	2.828377	1.148098
C	-3.942437	2.422438	-0.940239
H	-4.963063	2.122201	-0.680507
H	-3.985241	3.479542	-1.214379
C	-3.448500	1.591316	-2.125483
H	-4.110233	1.709552	-2.987418
H	-2.444992	1.895378	-2.438894
H	-3.412094	0.526250	-1.874976
H	4.662875	-2.815522	-0.518618
H	4.854453	-2.474777	1.201186
H	4.522974	-4.123036	0.659192
H	3.982817	-0.097825	0.112073

2k

C	-0.064183	-2.526496	-0.365684
C	0.418140	-3.824196	-0.564682
C	1.789949	-4.071436	-0.614410
C	2.743876	-3.049290	-0.475191
C	2.254491	-1.756170	-0.277844
C	0.864572	-1.509416	-0.223901
C	0.692347	-0.067853	0.006839
C	2.054340	0.511502	0.080037
C	2.961944	-0.481970	-0.089457
N	-0.444471	0.523268	0.125696
S	2.340084	2.225463	0.348508
C	0.664260	2.727683	0.497981
N	-0.412196	1.867311	0.353932
C	-1.528461	2.672148	0.550233
N	-1.138653	3.893884	0.784337
N	0.254341	3.933761	0.751323
H	-1.127084	-2.321429	-0.321323
H	-0.277789	-4.647292	-0.677204

H	2.136753	-5.089148	-0.763683
C	-2.941024	2.175976	0.450963
C	-3.816746	2.793375	1.552594
H	-2.905647	1.091278	0.589917
C	-3.512085	2.461110	-0.953245
H	-4.521080	2.048536	-1.040835
H	-3.562718	3.537980	-1.132060
H	-4.839698	2.416195	1.470551
H	-3.437772	2.544453	2.546844
H	-3.839710	3.882033	1.466456
H	-2.895241	2.013884	-1.737438
C	4.221246	-3.344196	-0.546903
H	4.406830	-4.419307	-0.514662
H	4.658647	-2.961317	-1.475280
H	4.766218	-2.886132	0.283691
H	4.036522	-0.364190	-0.086101

2l

C	0.374334	0.179063	2.429323
C	0.877774	0.258746	3.733716
C	2.250274	0.235956	3.964422
C	3.190052	0.134001	2.920454
C	2.681656	0.053821	1.624346
C	1.284854	0.076678	1.393913
C	1.084428	-0.026085	-0.059081
C	2.433349	-0.097550	-0.663649
C	3.360583	-0.054188	0.324076
N	-0.065781	-0.053685	-0.635423
S	2.681058	-0.212500	-2.399368
C	0.994915	-0.193652	-2.883273
N	-0.069825	-0.161858	-1.996171
C	-1.199834	-0.158579	-2.813243
N	-0.813056	-0.193990	-4.065026
N	0.569101	-0.215628	-4.111439
H	-0.691452	0.202114	2.235858
H	0.194929	0.341650	4.571305
H	2.612830	0.301821	4.985958
C	4.666993	0.102276	3.226419
C	-2.600497	-0.109093	-2.379691
C	-3.038680	-0.579999	-1.133726
C	-3.542736	0.398965	-3.289161
C	-4.392103	-0.535480	-0.806940
C	-5.321138	-0.026627	-1.711124
C	-4.890779	0.438218	-2.954638
H	-2.326836	-0.977696	-0.425904
H	-3.200499	0.754260	-4.251934
H	-4.720525	-0.906756	0.157229
H	-6.372946	0.006693	-1.450385
H	-5.606917	0.834700	-3.664636

H	4.945168	-0.839313	3.711826
H	5.275358	0.202029	2.326482
H	4.944620	0.913088	3.906606
H	4.431328	-0.091400	0.182878
